# Supplementary figures and images for: Extracellular vesicle release in an experimental ventilator-induced lung injury porcine model
Source: PLoS One. 2025 Apr 9;20(4):e0320144. doi: 10.1371/journal.pone.0320144 (PMC11981186; doi:10.1371/journal.pone.0320144)

# Extracellular vesicles in bronchoalveolar lavage fluid

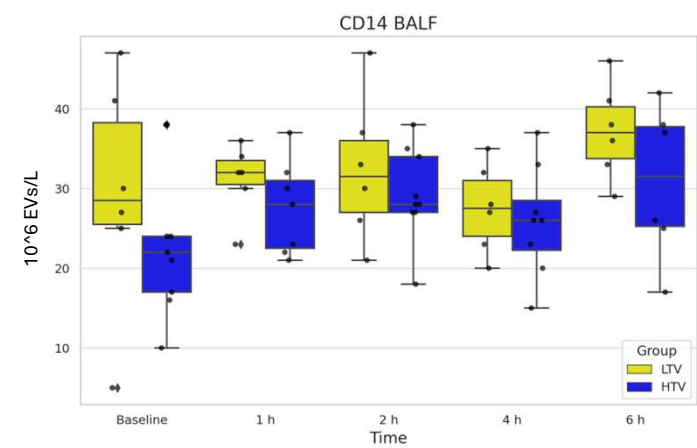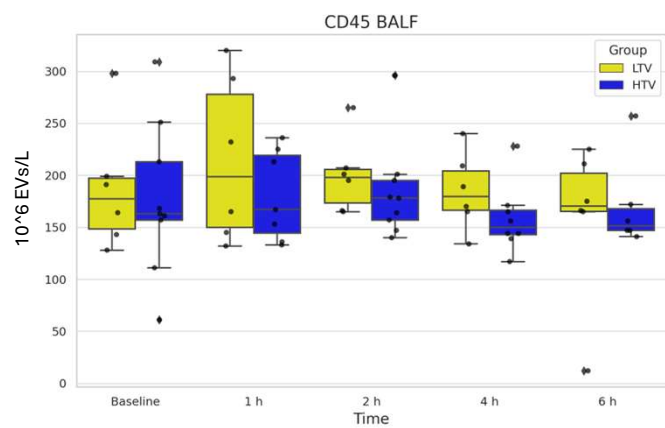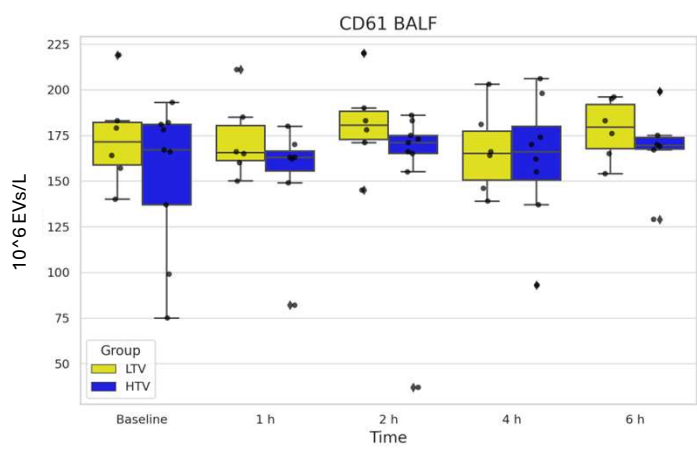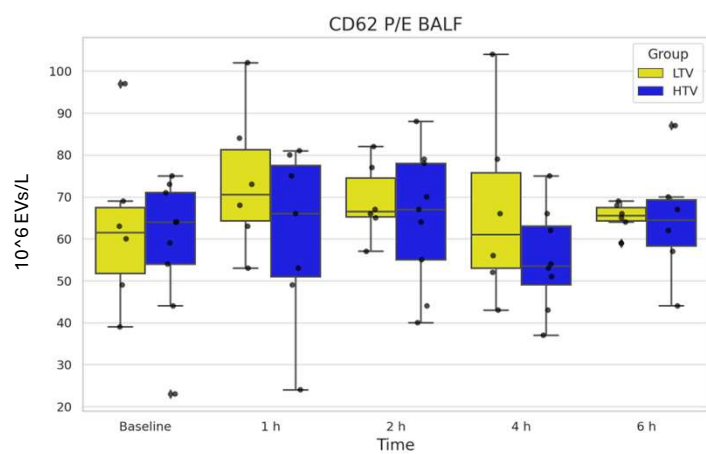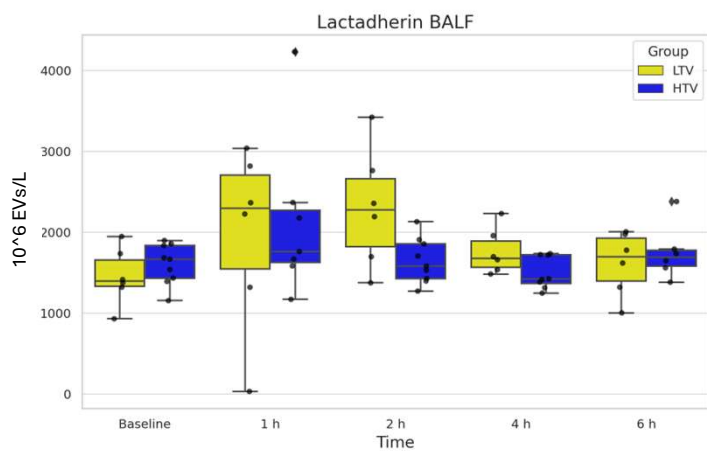

Supplement: S1 Fig — Panels of EVs positive for lactadherin and lactadherin in combination with CD14, CD45, CD61 and CD62P/E, respectively. (PDF) [file pone.0320144.s002.pdf]

# BALF Cells

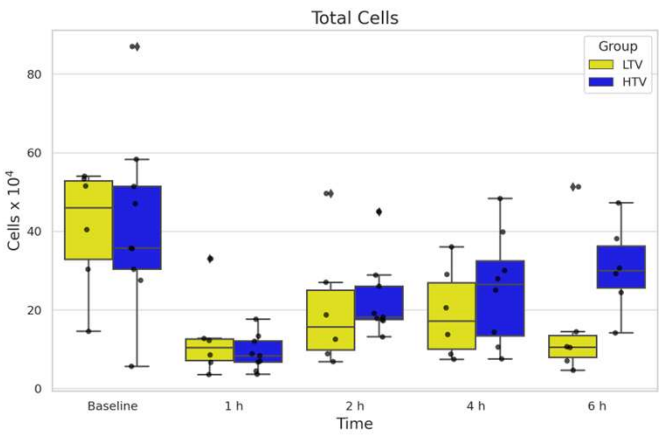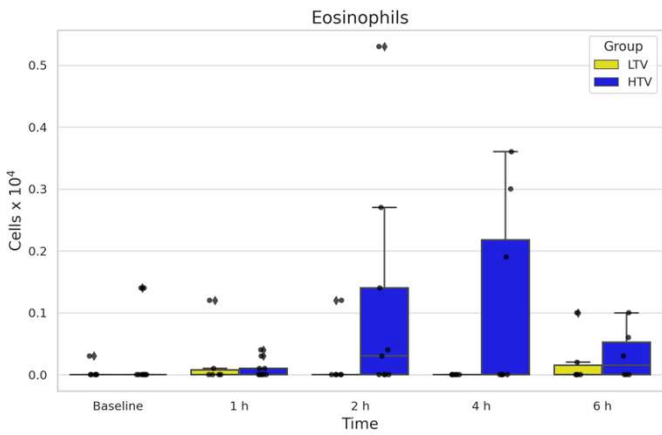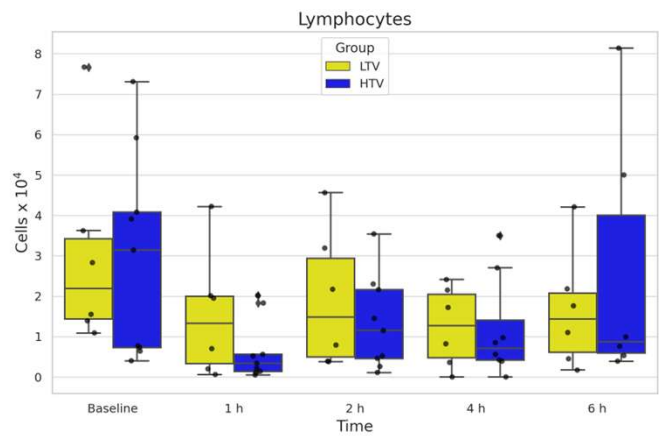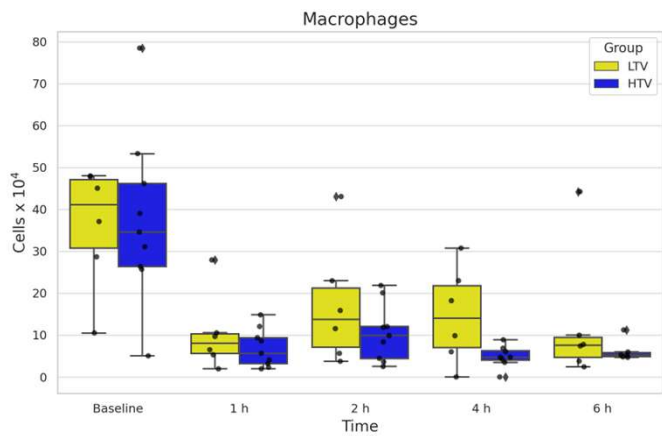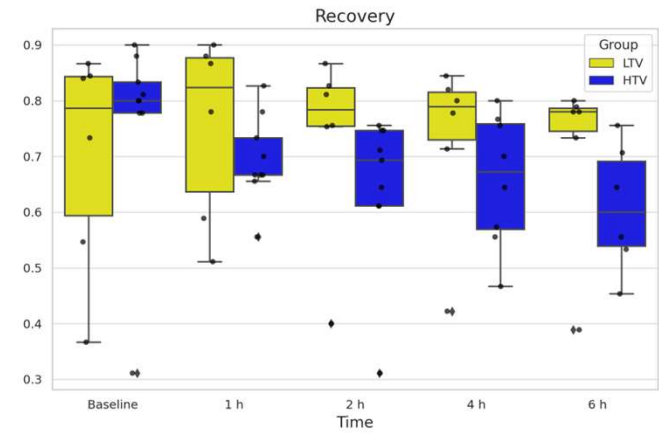

Supplement: S2 Fig — Cell counts of total cells, eosinophils, lymphocytes and macrophages as well as recovery rate. (PDF) [file pone.0320144.s003.pdf]

# Extracellular vesicles in plasma

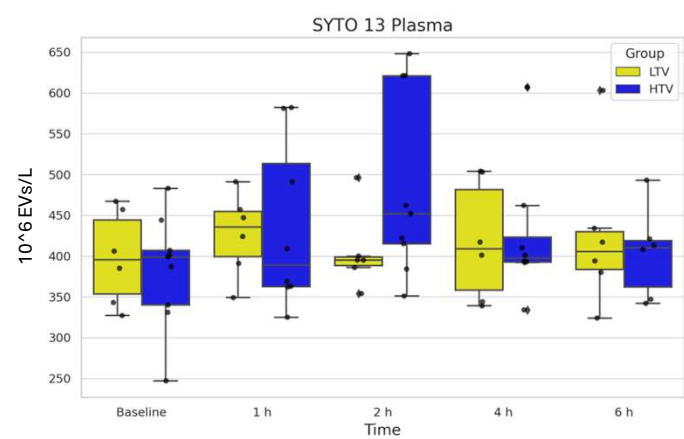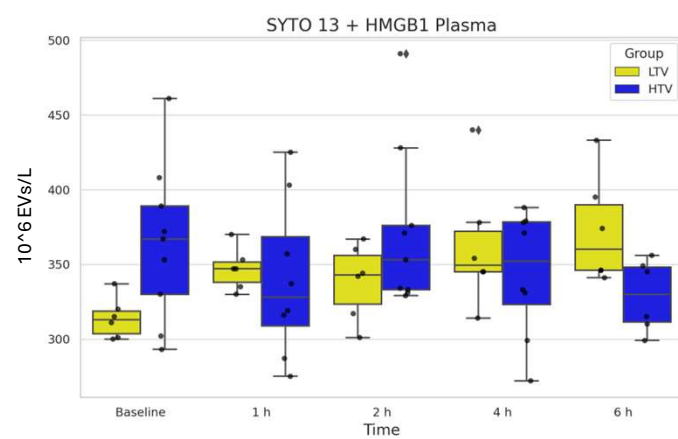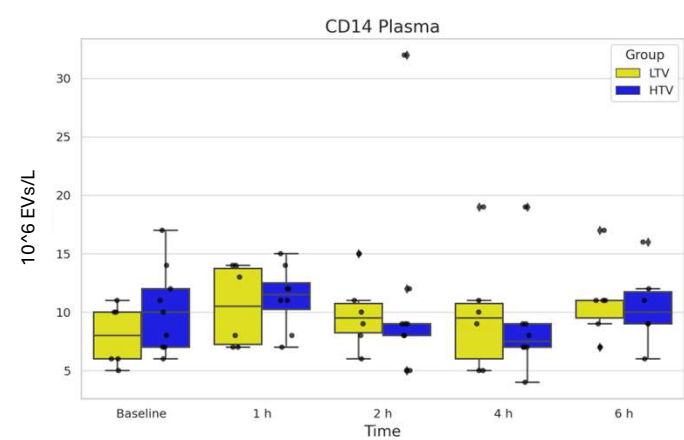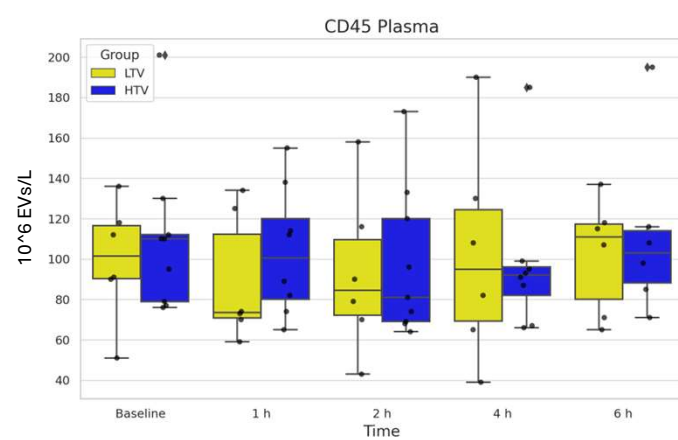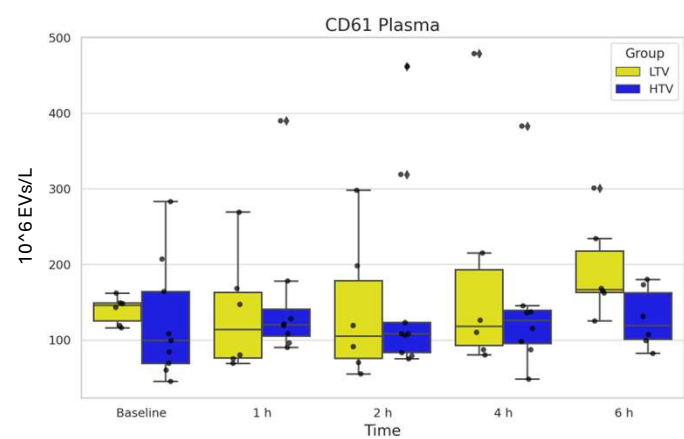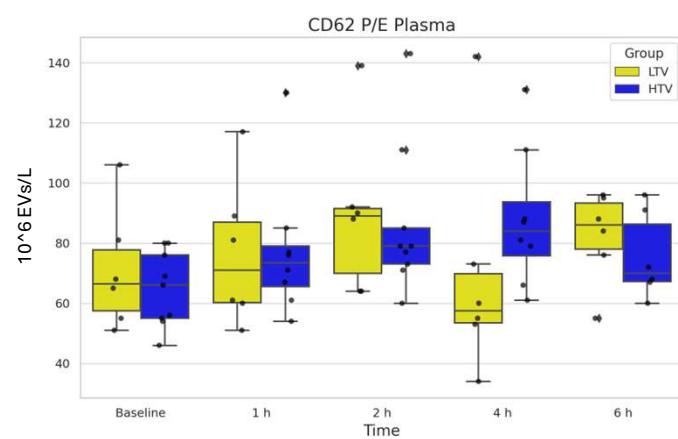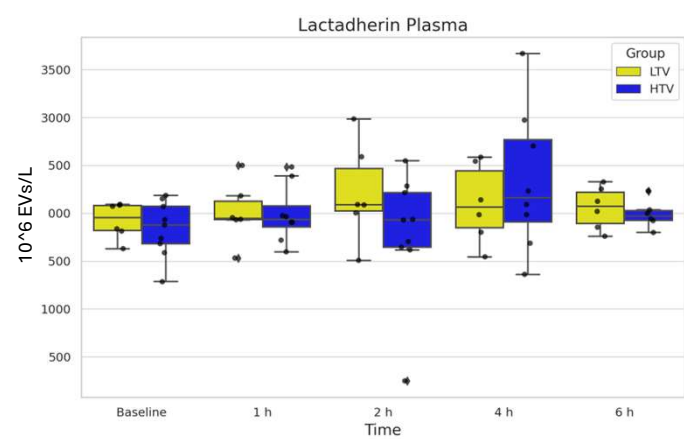

Supplement: S3 Fig — Panels of EVs positive for SYTO 13, SYTO 13 combined with HMGB1, lactadherin and lactadherin in combination with CD14, CD45, CD61 and CD62P/E, respectively. (PDF) [file pone.0320144.s004.pdf]
