# Supplementary material for: Extracellular vesicle release in an experimental ventilator-induced lung injury porcine model
Source: PLoS One. 2025 Apr 9;20(4):e0320144. doi: 10.1371/journal.pone.0320144 (PMC11981186; doi:10.1371/journal.pone.0320144)
Supplement: Attachment — Submitted filename: Response to reviewers.docx [file pone.0320144.s006.docx]

Thank you for your expert review and constructive comments. We have revised the manuscript accordingly and address all questions below. Our responses are marked in red text.

Journal Requirements:

When submitting your revision, we need you to address these additional requirements.

1. Please ensure that your manuscript meets PLOS ONE's style requirements, including those for file naming. The PLOS ONE style templates can be found at

https://journals.plos.org/plosone/s/file?id=wjVg/PLOSOne_formatting_sample_main_body.pdf and

https://journals.plos.org/plosone/s/file?id=ba62/PLOSOne_formatting_sample_title_authors_affiliations.pdf

The manuscript has been revised to confirm to the style templates and we have tried to catch all the details.

 2. Please include a caption for figure 1 and 5. Caption for figure 1 in included in rows 137-140. There is no figure 5 and the erroneous reference to fig 5 has been removed from the manuscript.

3. We note that the grant information you provided in the ‘Funding Information’ and ‘Financial Disclosure’ sections do not match.

When you resubmit, please ensure that you provide the correct grant numbers for the awards you received for your study in the ‘Funding Information’ section.

We have updated the financial disclosure statement. The organizations Västerbottens Läns Landsting and Umeå University have a cooperative grant arrangement and are linked in this context. We apologize for the previous unclarity.

4. Thank you for stating the following financial disclosure:

 [This work was supported by the ALF-LUA cooperative Region Västerbotten-Umeå University intramural research funding system.].

Please state what role the funders took in the study.  If the funders had no role, please state: ""The funders had no role in study design, data collection and analysis, decision to publish, or preparation of the manuscript.""

If this statement is not correct you must amend it as needed.

Please include this amended Role of Funder statement in your cover letter; we will change the online submission form on your behalf.

Done!

5. Thank you for stating the following in the Competing Interests section:

[Niklas Larsson has received speaker fees for lecturing in clinical symposia sponsored by Dräger Medical, unrelated to this specific topic.].

Please confirm that this does not alter your adherence to all PLOS ONE policies on sharing data and materials, by including the following statement: ""This does not alter our adherence to  PLOS ONE policies on sharing data and materials.” (as detailed online in our guide for authors http://journals.plos.org/plosone/s/competing-interests).  If there are restrictions on sharing of data and/or materials, please state these. Please note that we cannot proceed with consideration of your article until this information has been declared.

Please include your updated Competing Interests statement in your cover letter; we will change the online submission form on your behalf.

Done!
[Note: HTML markup is below. Please do not edit.]

Reviewers' comments:

Reviewer's Responses to Questions

**Comments to the Author**

1. Is the manuscript technically sound, and do the data support the conclusions?

The manuscript must describe a technically sound piece of scientific research with data that supports the conclusions. Experiments must have been conducted rigorously, with appropriate controls, replication, and sample sizes. The conclusions must be drawn appropriately based on the data presented.

Reviewer #1: Yes

Reviewer #2: No

2. Has the statistical analysis been performed appropriately and rigorously?

Reviewer #1: Yes

Reviewer #2: Yes

3. Have the authors made all data underlying the findings in their manuscript fully available?

The [PLOS Data policy](https://eur01.safelinks.protection.outlook.com/?url=http%3A%2F%2Ftrack.editorialmanager.com%2FCL0%2Fhttp%3A%252F%252Fwww.plosone.org%252Fstatic%252Fpolicies.action%2523sharing%2F1%2F010f0193cd931ede-6d00b2a9-e2eb-4de7-ab2b-b8c5dc215833-000000%2FXz2Z75pnN6XmNVzQCZItVuzoqhDq1y9QRMG6pe6icX0%3D189&data=05%7C02%7Cniklas.larsson%40umu.se%7C64f951ac0bf748311ec308dd1d843b76%7C5a4ba6f9f5314f329467398f19e69de4%7C0%7C0%7C638699176337234892%7CUnknown%7CTWFpbGZsb3d8eyJFbXB0eU1hcGkiOnRydWUsIlYiOiIwLjAuMDAwMCIsIlAiOiJXaW4zMiIsIkFOIjoiTWFpbCIsIldUIjoyfQ%3D%3D%7C0%7C%7C%7C&sdata=WE%2FfchW5I0dfnC12js%2BqqF4wEhUNfdO2PppWSFeAekU%3D&reserved=0) requires authors to make all data underlying the findings described in their manuscript fully available without restriction, with rare exception (please refer to the Data Availability Statement in the manuscript PDF file). The data should be provided as part of the manuscript or its supporting information, or deposited to a public repository. For example, in addition to summary statistics, the data points behind means, medians and variance measures should be available. If there are restrictions on publicly sharing data—e.g. participant privacy or use of data from a third party—those must be specified.

Reviewer #1: Yes

Reviewer #2: Yes

4. Is the manuscript presented in an intelligible fashion and written in standard English?

PLOS ONE does not copyedit accepted manuscripts, so the language in submitted articles must be clear, correct, and unambiguous. Any typographical or grammatical errors should be corrected at revision, so please note any specific errors here.

Reviewer #1: Yes

Reviewer #2: Yes

5. Review Comments to the Author

Please use the space provided to explain your answers to the questions above. You may also include additional comments for the author, including concerns about dual publication, research ethics, or publication ethics. (Please upload your review as an attachment if it exceeds 20,000 characters)

Reviewer #1: The authors have conducted an in-vivo study in pigs to evaluate how high tidal volume ventilation after surfactant depletion alters the generation of extracellular vessicles (EVs) in the bronchiolar lavage fluid and in the plasma. They also monitor a marker of lung damage and demonstrate that nucleic acid containing EVs increase in bronchoalveolar lavage fluid but not in the plasma. This is an important finding as it indicates that airspace markers might be more specific for lung injury during mechanical ventilation and that plasma biomarkers may not be indicated for assessing the degreee of lung injury during ventilation. This is an important study that I recommend for publication but I do have several comments that should be addressed prior to publication.

1. My main comment is that the ventilation protocol used, high tidal volume following lung lavage/surfactant inactivation may involve other forms of lung injury besides stretching injury. First, surfactant depletion will cause airway/alveolar atelectasis and even ventilation at the PEEP levels may result in cyclic airway closure and reopening. Recently, this form of injury (atelectrauma) has been implicated as a more damaging force than stretching, see Gabela-Zuniga, Lab on Chip, 2024. Second, the data in Table 2 indicate that there is higher inspiratory and airway pressures in the HTV groups and therefore transmural induced inflammation (barotrauma) may also be occuring. The authors should at minimum note the importance of these other forms of ventilation induced lung injury and note that they may be occuring to some degree in thier animal model. We agree in full and have added this to the discussion in rows 346-349.

2. I suggest referencing the novel work by Shaver et al in which protein biomarkers of ARDS were obtained from heat moisture exchange (HME) filter fluid as a way of assessing airway markers. Extension of this technique to obtain nucleic acids from HME fluid might be an important new way to non-invasively assess lung injury markers from the airspace and thus more specifically assess the degree of lung injury in mechanically ventilated patients See Bastarache, Am J Physiol Lung Cell Mol Physiol. 2021 and Rizzo, JCI Insight. 2022. . We agree that this is important. We have added a short reference to noninvasive sampling to the discussion in rows 328-329.

3. Since the authors use 100% O2 in the HTV group, there could also be hyperoxic lung injury. This should be noted. This is correct and we have added this to the discussion together with a comment on other possible injury mechanisms (row 346-349).

4. It should be noted that BAL could contain EVs from both airway as well as alveolar compartments. We agree and we have added this to the methods part (row 145-146) and to the discussion (row 294)

5. Given that neutrophil counts are going up in the presented model of VILI, it is possible that the increased EV counts seen in BAL are due to these cells. Is there any evidence for which cells these EVs come from? Surface markers?
In our study, the increased neutrophil counts in BAL support the hypothesis that the elevated EV levels could partially originate from these cells as you mention. While our study used markers like SYTO 13 and HMGB1 to indicate nucleic acid content and cellular stress, these are not specific for neutrophils. CD45, a general leukocyte marker included in the study, may also encompass EVs derived from neutrophils, though it lacks the specificity to confirm this conclusively. Neutrophil-specific markers such as CD66b or CD15 or MPO were not included. Future studies incorporating such markers would help definitively confirm the contribution of neutrophils to the EV pool observed in BAL. A short reference to this was added to the limitations paragraph in discussion (rows 358-361).

6. I think table 5 would be better served as a figure (like figure 3-4) . We believe the most relevant data is highlighted by limiting figures in the manuscript to the variables with significant changes in the intervention group. We have, however, included figures of all EVs and BALF cells in supplements to make overview easier. However, if the Editor wants us to, we will be happy to replace tables 4 and 5 with multi-panel figures like figure 3 and 4. Otherwise, we have updated supplements with S1-3 fig.

7. Finally, in addition to being a biomarker of disease, it might be worth mentioning that EVs have recently been used as a therapeutic drug delivery vehicle to minize VILI see Salazar‐Puerta, Advanced Materials, 2023. This is indeed a very interesting development. However, we feel that the scope of this paper is limited to biomarker and diagnostic aspects. Therapeutic aspects regarding VILI are possibly beyond the scope of the current paper.

Reviewer #2: Thank you for asking me to review this paper. Larssen et al use a porcine model of saline lung lavage and high stretch to examine release of extracellular vesicles in relation of VILI. They show increases in bronchoalveolar lavage neutrophils, and EVs containing SYTO-13 and HMGB-1 combined. I have a few comments:
1. I do not think the data add to the knowledge base. It is known that EVs and neutrophils are increased in ARDS/VILI. The main study question was if EVs, which appear in the lungs, in connection with acute lung injury, will be detectable in the first hours in plasma. We feel that this is a novel question and finding, although we cannot rule out plasma as a sampling source for early acute lung injury, our findings do not suggest that there is a large and rapid appearance of EVs from a specific organ injury in the early phase.

2. Further investigation as to where the EVs derived from would be useful, given the use of flow cytometry. We agree that further investigations of the EVs we studied would be very interesting. Unfortunately, we do not have more samples to analyze but EV parental cells in this context are very relevant for analysis in future studies.

3. Neutrophil EVs are associated with SYTO-13 especially if derived during NETosis induced DNA release.

While NETosis in this model is possible, we have not studied it specifically. SYTO-13 can indeed stain EVs associated with NETosis due to its binding to extracellular DNA. However, it is not specific to NETs or neutrophils, as it also binds to EVs from other cell types. In our previous studies, we have observed that platelets and platelet derived EVs exhibit strong SYTO-13 binding, likely due to their mRNA content. While the current findings could partially reflect neutrophil activity during NETosis, further studies specifically targeting neutrophil EVs and NETosis markers are needed to clarify this.

(Ref: <https://pubmed.ncbi.nlm.nih.gov/31103269/> )

4. HMGB-1 is also associated with NET release

HMGB-1 is indeed associated with NET release and could reflect neutrophil activity in this context. However, it is not specific to NETs or neutrophils, as HMGB-1 is also released by other cell types, including platelets, as documented in the literature. While HMGB1 is associated with cellular stress or damage, it cannot solely be attributed to NETosis or neutrophils. Future studies using more specific NET and neutrophil markers would be essential to better delineate the role of HMGB-1 in this context.

5. Increased neutrophil numbers in the BAL would also mean higher EVs being detected. Especially given other EVs e.g. platelet derived ones did not increase.

The increased neutrophil numbers in BAL likely contribute to the higher EV levels observed. However, it would be too speculative to definitively attribute the observed EV increase solely to neutrophils without the use of additional specific markers. Platelets are relevant as possible parent cells.

6. What are the downstream effects of the EVs that have been detected?
In the given context, any statement about downstream effects would be speculative, as this study does not analyze or establish functional effects of the EVs observed here. However, it is well-documented that EVs can have both pro-inflammatory and anti-inflammatory effects, depending on their cargo and other characteristics, which includes a variety of proteins, nucleic acids, and other bioactive molecules. These effects could influence inflammation, immune modulation, and tissue remodeling. Determining the specific downstream effects of the EVs observed in this study would require additional experimental approaches, such as functional assays or proteomic and transcriptomic analyses of EV cargo and pathway analysis.
